# Whole-exome sequencing reveals *POLR3B* variants associated with progeria-related Wiedemann-Rautenstrauch syndrome

**DOI:** 10.1186/s13052-021-01112-6

**Published:** 2021-07-21

**Authors:** Shao-Wen Wu, Lin Li, Fan Feng, Li Wang, Yuan-Yuan Kong, Xiao-Wei Liu, Chenghong Yin

**Affiliations:** 1 Department of Obstetrics, Beijing Obstetrics and Gynecology Hospital, Capital Medical University, Beijing, 100026 Chaoyang China; 2 Beijing Maternal and Child Health Care Hospital, Beijing, 100026 Chaoyang China; 3 Central Laboratory, Beijing Obstetrics and Gynecology Hospital, Capital Medical University, Beijing, 100026 Chaoyang China; 4 Department of Basic Medical Sciences, School of Medicine, Tsinghua University, Haidian, Beijing, 100084 China; 5 Department of Ultrasound, Beijing Obstetrics and Gynecology Hospital, Capital Medical University, Beijing, 100026 Chaoyang China; 6 Department of Newborn Screening, Beijing Obstetrics and Gynecology Hospital, Capital Medical University, Beijing, 100026 Chaoyang China

**Keywords:** Wiedemann-Rautenstrauch syndrome, Whole-exome sequencing, POLR3B, Progeria, Growth retardation

## Abstract

**Background:**

Wiedemann-Rautenstrauch syndrome (WRS) is a rare autosomal recessive neonatal progeroid disorder characterized by prenatal and postnatal growth retardation, short stature, a progeroid appearance, hypotonia, and mental impairment.

**Case presentation:**

A 6-year-old patient, who initially presented with multiple postnatal abnormalities, facial dysplasia, micrognathia, skull appearance, hallux valgus, and congenital dislocation of the hip, was recruited in this study. The patient was initially diagnosed with progeria. The mother of the patient had abnormal fetal development during her second pregnancy check-up, and the clinical phenotype of the fetus was similar to that of the patient. Whole-exome sequencing (WES) of the patient was performed, and *POLR3B* compound heterozygous variants—c.2191G > C:p.E731Q and c.3046G > A:p.V1016M—were identified in the patient. Using Sanger sequencing, we found that the phenotypes and genotypes were segregated within the pedigree. These two variants are novel and not found in the gnomAD and 1000 Genomes databases. The two mutation sites are highly conserved between humans and zebrafish.

**Conclusions:**

Our study not only identified a novel WRS-associated gene, *POLR3B*, but also broadened the mutational and phenotypic spectra of *POLR3B*. Furthermore, WES may be useful for identifying rare disease-related genetic variants.

**Supplementary Information:**

The online version contains supplementary material available at 10.1186/s13052-021-01112-6.

## Background

Progeria, also known as premature aging syndrome, accelerates the aging of newborns. Characteristic facial features include a larger head, narrow nose bridge, narrow nasal tip, thin vermilion of the upper and lower lips, and a small mouth [1, 2]. Common symptoms include decreased subcutaneous fat, delayed eruption and loss of primary teeth, abnormal skin on the abdomen and upper thighs, early hair loss, nail dystrophy, hip valgus, and decreased joint mobility [1, 2]. The mean age at diagnosis is 2.9 years [1]. The average age of demise for patients with progeria is approximately 12.6 years [1]. Hutchinson-Gilford Progeria is an accelerated aging syndrome caused by mutations in *LMNA* [3].

Wiedemann-Rautenstrauch syndrome (WRS; OMIM:264090) is a rare autosomal recessive neonatal progeroid disorder that includes some features of premature aging, but it is not caused by laminopathy. WRS is characterized by prenatal and postnatal growth retardation, short stature, a progeroid appearance (sparse scalp hair, lipodystrophy, triangular face, pointed chin, and natal teeth), hypotonia, and mental impairment [4]. WRS is mainly caused by biallelic mutations in *POLR3A* [5–8]. Recently, a homozygous nonsense variant of *POLR3GL* has been found to be associated with WRS [9].

Here, we report a proband with WRS. Whole-exome sequencing (WES) of the proband was performed, and compound heterozygous sequence variants of *POLR3B* were identified. Thus, in addition to previously reported mutations in *POLR3A* and *POLR3GL*, in the present study, we identified a novel gene, *POLR3B*, related to the pathogenesis of WRS.

## Case presentation

**Fig. 1:**
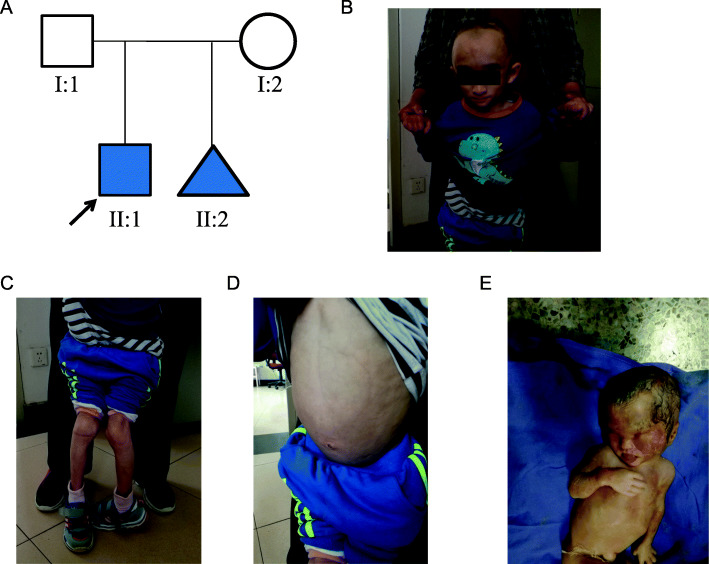
Pedigree analysis and images of the proband with Wiedemann-Rautenstrauch Syndrome. **A** The family tree shows the proband (II:1) from a non-consanguineous family. II:2 is the fetus from induced labor. **B** Facial photograph of the proband at 6 years of age. Physical features include macrocephaly, sparse scalp hair, prominent scalp veins, broad forehead, triangular face, pointed chin, thin upper lip vermilion, and abnormality of the ear. **C** Photograph of the leg bones of the proband. Physical features include abnormality of the skeletal system and loss of subcutaneous fat from the extremities. **D** Photograph of the abdomen of the proband. Physical features include lipodystrophy and extreme emaciation. **E** Photograph of the abortus. Physical features include abnormal head

The proband was a 6-year-old boy. At birth, his weight was 2200 g and length was 50 cm, and his parents were not consanguineous (Fig. 1A). No abnormality was found in the proband during the pregnancy examination of the mother. The proband had multiple postnatal abnormalities, facial dysplasia, micrognathia, skull appearance, hallux valgus, and congenital dislocation of the hip (Fig. 1B-D). The clinical diagnosis was progeria. The mother of the proband underwent regular prenatal examinations during her second pregnancy. Due to being a “pregnant woman in older age, with poor pregnancy history,” the mother of the proband underwent amniocentesis and genetic examination, and no abnormalities were found in the fetus. No abnormality was detected in the fetus following ultrasound at 23 weeks of gestation. The 29-week ultrasound showed that the fetus had a “lemon head”. Magnetic resonance imaging (MRI) of the fetal head showed that the fetal brain was “lemon-shaped” and that the fetal frontal lobe was relatively small. We arranged a pediatric neurosurgery consultation, and the consultation doctors considered the possibility of disability after birth. After discussing with the family and the hospital obstetrician, it was decided to induce labor. The pregnant woman gave birth to a dead girl weighing 1590 g, and the dead girl had abnormal head development (Fig. 1E).

We first analyzed the pedigree (Fig. 1A) and considered that the disease might have a recessive mode of inheritance. First, CNV sequencing was performed to assess whether there were any CNV perturbations in II:1. We did not identify any chromosome aneuploidy variations or known microdeletion/microduplication variants over 100 kb in II:1 (Supplementary Figure 1A). Therefore, WES was used to determine the genetic causes of II:1. The quality metrics for coverage of the target regions of the exome were satisfactory (Supplementary Table 1). When we analyzed the WES data, we prioritized homozygous variants or compound heterozygous variants. We filtered out the variants with allele frequencies greater than 1% in the databases, including the 1000 Genomes database (1000G, http://browser.1000genomes.org/index.html), the Short Genetic Variations database (dbSNP, http://www.ncbi.nlm.nih.gov/snp/), the Exome Variant Server (ESP6500, http://evs.gs.washington.edu/EVS/), and an in-house database (the retained variants are listed in Supplementary Table 2). Next, we considered that both the proband and aborted fetus had the phenotype of progeria’ therefore, we prioritized the analysis of gene mutations related to progeria, such as in *LMNA* [3, 10]. However, we did not find any potential pathogenic variants of *LMNA* in the proband. We also focused on the pathogenic genes of other diseases related to progeria, such as *POLR3A* [5, 6] and *POLR3GL* [9], and we only found a heterozygous *POLR3A* variant, c.1771-6C > A, which is an intron variant, in the WES data. Since the biallelic mutations of *POLR3A* were pathogenic, the heterozygous intron variant that we found may not be related to the occurrence of the disease. However, we found that the proband carried compound heterozygous variants (NM_018082.5:c.2191G > C:p.E731Q and c.3046G > A:p.V1016M) of *POLR3B*, which encodes a protein whose function is closely related to that of POLR3A.

**Fig. 2:**
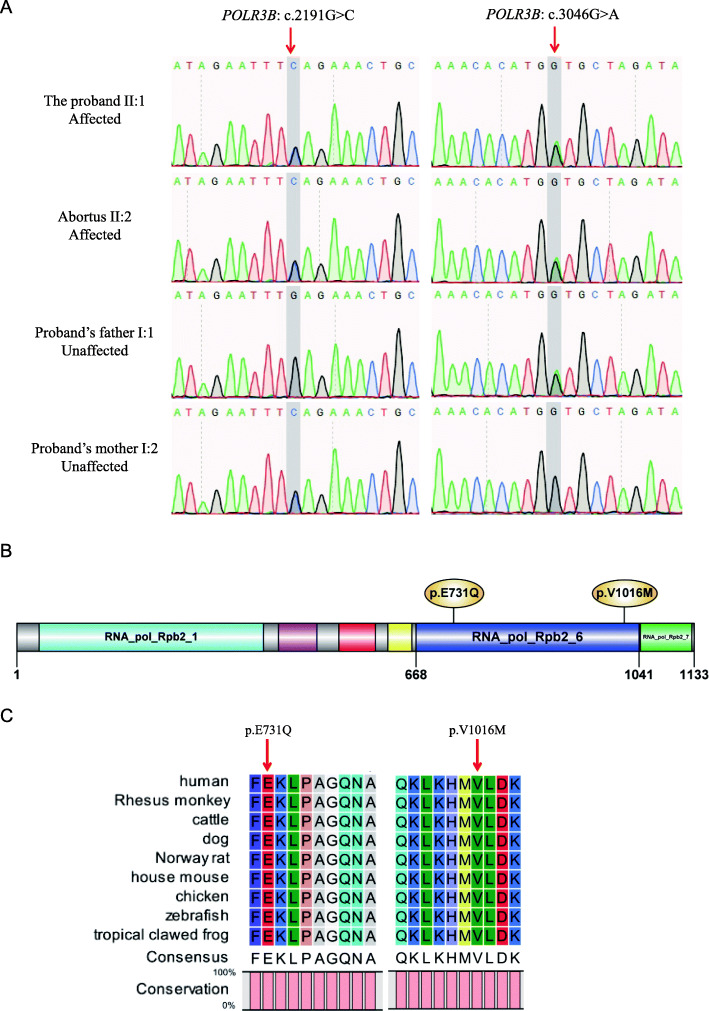
Validation and analysis of *POLR3B* variants. **A** Sanger sequencing confirmed the compound heterozygous *POLR3B* variants in the proband and abortus. The father and mother of the porband each carried a heterozygous allele. The red arrows indicate the variant sites. **B** Domains and variant sites in POLR3B. The full-length protein comprises 1133 amino acids. RNA_pol_Rpb2_1 domain (light blue box); RNA_pol_Rpb2_3 domain (purple box); RNA_pol_Rpb2_4 domain (red box); RNA_pol_Rpb2_5 domain (yellow box); RNA_pol_Rpb2_6 domain (dark blue box); RNA_pol_Rpb2_7 domain (green box). **C** Sequence alignment of POLR3B in different species. The red arrows indicate the variant sites

Sanger sequencing was used to validate the variants in each member of the pedigree. Both the proband and aborted fetus harbored two variants (Fig. 2A), of which c.2191G > C was inherited from the mother (Fig. 2A) and c.3046G > A was inherited from the father (Fig. 2A).

To understand the pathogenicity of the two variants, we first conducted bioinformatics analysis. Using online prediction tools, including Polyphen-2, SIFT, PROVEAN, and Mutation Taster, the two variants were considered pathogenic or damaging mutations (Table 1). The predicted results were also supported by the very low allele frequencies of the two mutations in the 1000G and gnomAD databases (Table 1). In addition, we analyzed the conservation of the two variants. Both variants were located in the RNA_pol_Rpb2_6 domain of POLR3B (Fig. 2B). The results of protein sequence alignment analysis revealed that the mutation sites were 100% conserved from humans to zebrafish (Fig. 2B), which indicated that the amino acids at the variant sites were irreplaceable in evolutionary history. The American College of Medical Genetics and Genomics guidelines predicted the two variants as variants of uncertain significance (Table 1).

**Table 1:** Bioinformatic analysis of the *POLR3B* variants

Variant	Amino acid change	Polyphen-2^a^	SIFT^b^	PROVEAN^c^	Mutation Taster^d^	ACMG^e^	1000G^f^	gnomAD (total)^g^
c.2191G > C	p.E731Q	Possibly damaging (0.828)	Damaging (0.002)	Neutral (−2.02)	Disease causing (1.0)	VUS	0	0
c.3046G > A	p.V1016M	Probably damaging (1.000)	Damaging (0.000)	Damaging (−2.91)	Disease causing (1.0)	VUS	0	0

^a^Polyphen-2. Prediction Scores range from 0 to 1 with high scores indicating probably or possibly damaging

^b^SIFT, i.e., Sorting Intolerant From Tolerant. Scores vary between 0 and 1. Variants with scores close or equal to 0 are predicted to be damaging

^c^PROVEAN. Variants with scores lower than − 2.5 (cutoff) are predicted to be deleterious

^d^Mutation Taster. The probability value is the probability of the prediction, i.e., a value close to 1 indicates a high ‘security’ of the prediction

^e^American College of Medical Genetics and Genomics/Association for Molecular Pathology (ACMG/AMP) variant classification. VUS, variants of uncertain significance

^f^Allele frequency of variation in 1000 Genomes (1000G) database

^g^Allele frequency of variation in total of gnomAD (genome Aggregation Database, a big database containing 123,136 exome sequences and 15,496 whole-genome sequences)

**Fig. 3:**
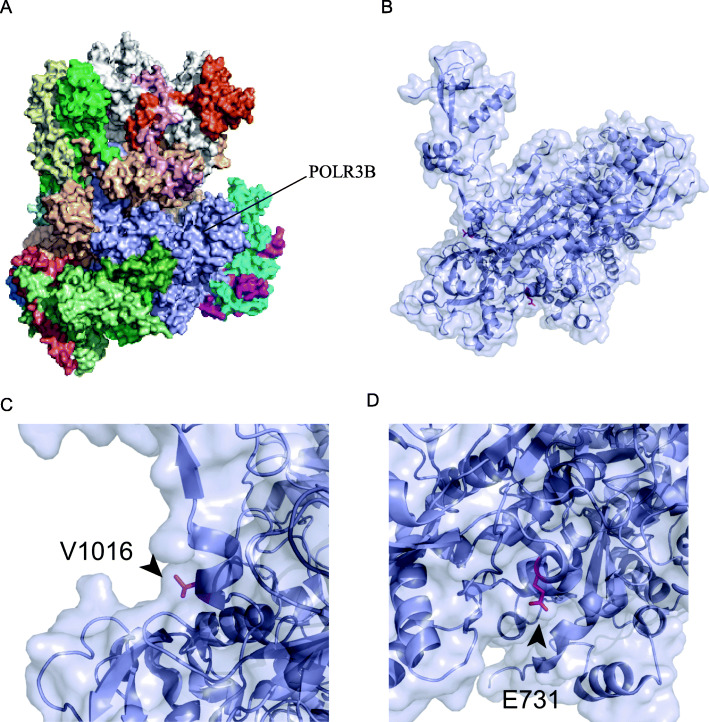
POLR3B structure and mutation site analysis. **A** Protein complex structure of RNA polymerase III. **B** POLR3B structure is shown as a cartoon. V1016 and E731 side chains are shown as sticks. **C** Detailed structural information of the V1016 residue. **D** Detailed structural information of the E731 residue

We further analyzed the possible pathogenicity of the variant sites by analyzing the structure of POLR3B. The structures of RNA polymerase III and POLR3B are both known (Fig. 3A). POLR3B (Fig. 3B), which is an important subunit of RNA polymerase III, forms the catalytic active center of the polymerase together with POLR3A. The V1016 residue is very important (Fig. 3C) because previous studies have shown that the H1014 and K1019 residues, which are very close to the V1016 residue, are the amino acids that directly interact with nucleotides in POLR3B. Recent findings suggest that the K1019 residue interacts with the template DNA strand and that the H1014 residue interacts with the newly transcribed RNA strand [11]. Therefore, we speculated that V1016M might affect the catalytic activity of RNA polymerase III. The location of the E731 residues provides us with insufficient information regarding the mechanistic processes (Fig. 3D), and more experimental evidence is needed to clarify the involvement of the residues in the pathogenesis of the disease.

## Discussion and conclusions

In this study, a patient with progeria phenotype underwent WES, and we found that the patient carried compound heterozygous variants of the *POLR3B* gene. Based on the genetic variants and the clinical phenotype of the patient, we believe that the patient should be diagnosed with WRS. The genes associated with WRS pathogenesis, identified in previous studies, are *POLR3A* and *POLR3GL* [6, 9]. Therefore, *POLR3B* identified in the present study is a novel pathogenic gene for WRS.

The most prominent feature of WRS is progeria. The clinical phenotype of the proband in this study was progeria; thus, we focused on progeria-related genes in the initial WES analysis. However, we did not find any mutations in *LMNA*. The advantage of WES over gene panel detection is that it can cover variants of almost all protein-coding genes. Therefore, we began to search for other genetic variants when it was determined that the patient was *LMNA* mutation-negative. Independent genetic analysis identified compound heterozygous variants of *POLR3B*. *POLR3A* variants, which encode proteins closely related to POLR3B in function, can lead to progeria-related WRS. Accordingly, we further confirmed the possibility of *POLR3B* biallelic variants leading to WRS.

*POLR3B* is located in the chr 12q23.3 region and consists of 30 exons. *POLR3A* and *POLR3B* encode for the largest (RPC1) and second largest (RPC2) subunits of RNA polymerase III, respectively, which are required for the transcription of a subset of non-protein-coding RNAs that includes 5S ribosomal RNA and transfer RNAs (tRNAs) [12]. POLR3A and POLR3B together form the catalytic active center of the polymerase and contribute to the catalytic activity of the polymerase [13]. Therefore, it is reasonable to consider that *POLR3A* and *POLR3B* mutations cause similar phenotypes. Previous studies have found that *POLR3B* mutations can cause hypomyelination, hypodontia, and hypogonadotropic hypogonadism (also known as 4H syndrome) [14–16]. We believe that *POLR3B* mutations that cause 4H syndrome do not interfere with the mutations that cause WRS because numerous studies have reported the mutational spectrum of *POLR3A*; although biallelic *POLR3A* mutations have been previously reported in patients with 4H syndrome or spastic ataxia, haplotypes have been detected in WRS patients. Even though there are major differences between 4H and WRS, there is some overlap in disease phenotypes, suggesting that mutations in *POLR3A* and *POLR3B* might be related to both diseases. Reduction in the levels of *POLR3A* or *POLR3B* leads to perturbation of transcription of the total pool of tRNAs and a deregulated transcription of certain types of ncRNAs [17, 18], could be a molecular mechanism underlying *POLR3A* or *POLR3B* mutations. Recently, a sequence variant of *POLR3GL* has been reported to be associated with WRS [9]. *POLR3GL* encodes one of the 17 subunits of RNA polymerase III. POLR3GL (RPC32) forms a stable subcomplex together with POLR1C (RPC62) and POLR3F (RPC39). The subcomplex directs RNA polymerase III binding to the TFIIIB-DNA complex via interactions between TFIIIB and POLR3F [19]. Therefore, these results suggest that perturbations in *POLR3A*, *POLR3B*, and *POLR3GL*, which encode three subunits of RNA polymerase III, might be associated with WRS.

In summary, this study not only identified a novel WRS-associated gene, *POLR3B*, but also enriched the mutational and disease spectra of *POLR3B*. Furthermore, we believe that next-generation sequencing technology could be very useful for identifying rare disease-related genetic mutations.

## Supplementary Information

**Additional file 1: Table S1.** Statistics of the WES data.

**Additional file 2.**

**Additional file 3.**

## Data Availability

The datasets used and/or analyzed during the current study are available from the corresponding author upon reasonable request.

## References

[1] Hennekam RC (2006). Hutchinson-Gilford progeria syndrome: review of the phenotype. Am J Med Genet A.

[2] Foo MXR, Ong PF, Dreesen O (2019). Premature aging syndromes: from patients to mechanism. J Dermatol Sci.

[3] De Sandre-Giovannoli A, Bernard R, Cau P, Navarro C, Amiel J, Boccaccio I (2003). Lamin a truncation in Hutchinson-Gilford progeria. Science.

[4] Paolacci S, Bertola D, Franco J, Mohammed S, Tartaglia M, Wollnik B, Hennekam RC (2017). Wiedemann-Rautenstrauch syndrome: a phenotype analysis. Am J Med Genet A.

[5] Jay AM, Conway RL, Thiffault I, Saunders C, Farrow E, Adams J, Toriello HV (2016). Neonatal progeriod syndrome associated with biallelic truncating variants in POLR3A. Am J Med Genet A.

[6] Wambach JA, Wegner DJ, Patni N, Kircher M, Willing MC, Baldridge D, Xing C, Agarwal AK, Vergano SAS, Patel C, Grange DK, Kenney A, Najaf T, Nickerson DA, Bamshad MJ, Cole FS, Garg A (2018). Bi-allelic POLR3A loss-of-function variants cause autosomal-recessive Wiedemann-Rautenstrauch syndrome. Am J Hum Genet.

[7] Lessel D, Ozel AB, Campbell SE, Saadi A, Arlt MF, McSweeney KM (2018). Analyses of LMNA-negative juvenile progeroid cases confirms biallelic POLR3A mutations in Wiedemann-Rautenstrauch-like syndrome and expands the phenotypic spectrum of PYCR1 mutations. Hum Genet.

[8] Paolacci S, Li Y, Agolini E, Bellacchio E, Arboleda-Bustos CE, Carrero D, Bertola D, al-Gazali L, Alders M, Altmüller J, Arboleda G, Beleggia F, Bruselles A, Ciolfi A, Gillessen-Kaesbach G, Krieg T, Mohammed S, Müller C, Novelli A, Ortega J, Sandoval A, Velasco G, Yigit G, Arboleda H, Lopez-Otin C, Wollnik B, Tartaglia M, Hennekam RC (2018). Specific combinations of biallelic POLR3A variants cause Wiedemann-Rautenstrauch syndrome. J Med Genet.

[9] Beauregard-Lacroix E, Salian S, Kim H, Ehresmann S, D'Amours G, Gauthier J (2020). A variant of neonatal progeroid syndrome, or Wiedemann-Rautenstrauch syndrome, is associated with a nonsense variant in POLR3GL. Eur J Hum Genet.

[10] Gonzalo S, Kreienkamp R, Askjaer P (2017). Hutchinson-Gilford progeria syndrome: a premature aging disease caused by LMNA gene mutations. Ageing Res Rev.

[11] Girbig M, Misiaszek AD, Vorländer MK, Lafita A, Grötsch H, Baudin F, Bateman A, Müller CW (2021). Cryo-EM structures of human RNA polymerase III in its unbound and transcribing states. Nat Struct Mol Biol.

[12] Sepehri S, Hernandez N (1997). The largest subunit of human RNA polymerase III is closely related to the largest subunit of yeast and trypanosome RNA polymerase III. Genome Res.

[13] Werner M, Thuriaux P, Soutourina J (2009). Structure-function analysis of RNA polymerases I and III. Curr Opin Struct Biol.

[14] Saitsu H, Osaka H, Sasaki M, Takanashi J, Hamada K, Yamashita A, Shibayama H, Shiina M, Kondo Y, Nishiyama K, Tsurusaki Y, Miyake N, Doi H, Ogata K, Inoue K, Matsumoto N (2011). Mutations in POLR3A and POLR3B encoding RNA polymerase III subunits cause an autosomal-recessive hypomyelinating leukoencephalopathy. Am J Hum Genet.

[15] Wolf NI, Vanderver A, van Spaendonk RM, Schiffmann R, Brais B, Bugiani M (2014). Clinical spectrum of 4H leukodystrophy caused by POLR3A and POLR3B mutations. Neurology.

[16] Verberne EA, Dalen Meurs L, Wolf NI, van Haelst MM (2020). 4H leukodystrophy caused by a homozygous POLR3B mutation: further delineation of the phenotype. Am J Med Genet A.

[17] Azmanov DN, Siira SJ, Chamova T, Kaprelyan A, Guergueltcheva V, Shearwood AJ (2016). Transcriptome-wide effects of a POLR3A gene mutation in patients with an unusual phenotype of striatal involvement. Hum Mol Genet.

[18] Yee NS, Gong W, Huang Y, Lorent K, Dolan AC, Maraia RJ, Pack M (2007). Mutation of RNA pol III subunit rpc2/polr3b leads to deficiency of subunit Rpc11 and disrupts zebrafish digestive development. PLoS Biol.

[19] Wang Z, Roeder RG (1997). Three human RNA polymerase III-specific subunits form a subcomplex with a selective function in specific transcription initiation. Genes Dev.

