# Supplementary material for: Whole-exome sequencing reveals POLR3B variants associated with progeria-related Wiedemann-Rautenstrauch syndrome
Source: Ital J Pediatr. 2021 Jul 21;47:160. doi: 10.1186/s13052-021-01112-6 (PMC8296688; doi:10.1186/s13052-021-01112-6)
Supplement: Supplementary file 1 — Additional file 1: Table S1. Statistics of the WES data. [file 13052_2021_1112_MOESM1_ESM.docx]

**Supplementary Table 1.** Statistics of the WES data

| **Sample** | **II:1** |
| --- | --- |
| Raw data (Mb) | 18187.26 |
| Target Region (bp) | 58,682,415 |
| Coverage of Target Region | 99.88% |
| Mean Depth of Target Region (X) | 135.78 |
| Fraction of Target Covered >4X | 99.76% |
| Fraction of Target Covered >10X | 99.44% |
| Fraction of Target Covered >30X | 97.22% |
