# Supplementary figures and images for: Whole-exome sequencing reveals POLR3B variants associated with progeria-related Wiedemann-Rautenstrauch syndrome
Source: Ital J Pediatr. 2021 Jul 21;47:160. doi: 10.1186/s13052-021-01112-6 (PMC8296688; doi:10.1186/s13052-021-01112-6)

A

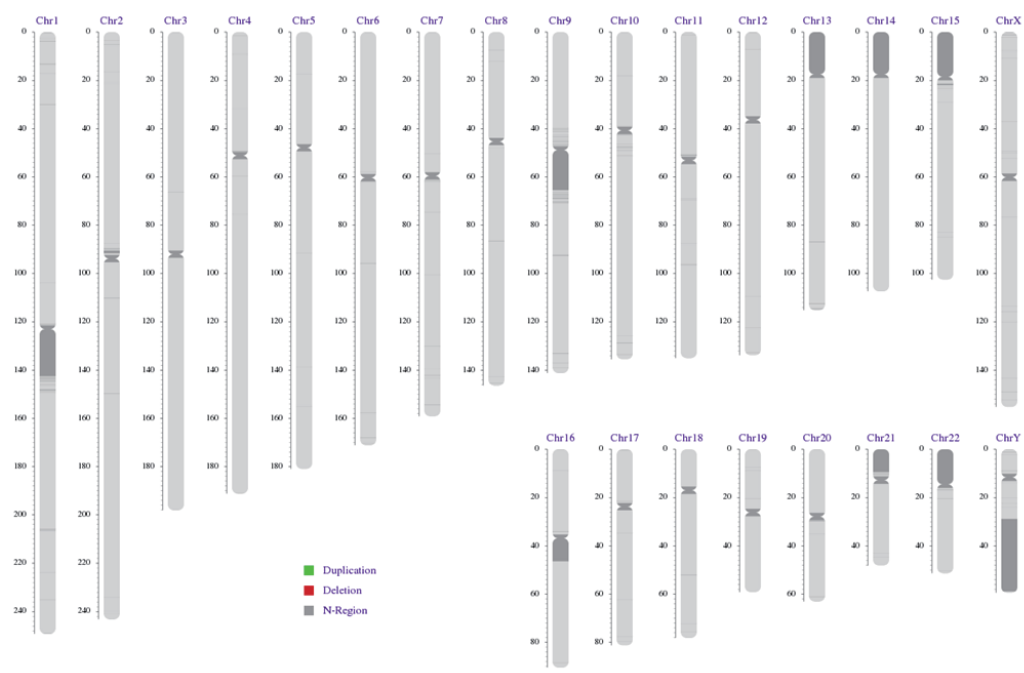

Supplement: Supplementary file 3 — Additional file 3. [file 13052_2021_1112_MOESM3_ESM.pdf]
